# The distribution of carotenoids in hens fed on biofortified maize is influenced by feed composition, absorption, resource allocation and storage

**DOI:** 10.1038/srep35346

**Published:** 2016-10-14

**Authors:** Jose Antonio Moreno, Joana Díaz-Gómez, Carmina Nogareda, Eduardo Angulo, Gerhard Sandmann, Manuel Portero-Otin, José C. E. Serrano, Richard M. Twyman, Teresa Capell, Changfu Zhu, Paul Christou

**Affiliations:** 1 Department of Animal Science, ETSEA, University of Lleida-Agrotecnio Center, Av. Alcalde Rovira Roure, 191, 25198 Lleida, Spain; 2 Department of Food Technology, ETSEA, University of Lleida-Agrotecnio Center, Av. Alcalde Rovira Roure, 191, 25198 Lleida, Spain; 3 Biosynthesis Group, Department of Molecular Biosciences, J. W. Goethe University, Max-v-Laue Str. 9, D-60438 Frankfurt, Germany; 4 Department of Experimental Medicine, Faculty of Medicine, University of Lleida–Institut de Recerca Biomèdica de Lleida (IRBLleida), Av. Rovira Roure 80, 25198 Lleida, Spain; 5 TRM Ltd, PO Box 463, York, YO11 9FJ, United Kingdom; 6 Department of Plant Production and Forestry Science, ETSEA, University of Lleida-Agrotecnio Center, Av. Alcalde Rovira Roure, 191, 25198 Lleida, Spain; 7 ICREA, Catalan Institute for Research and Advanced Studies, Passeig Lluís Companys 23, 08010 Barcelona, Spain

## Abstract

Carotenoids are important dietary nutrients with health-promoting effects. The biofortification of staple foods with carotenoids provides an efficient delivery strategy but little is known about the fate and distribution of carotenoids supplied in this manner. The chicken provides a good model of human carotenoid metabolism so we supplemented the diets of laying hens using two biofortified maize varieties with distinct carotenoid profiles and compared the fate of the different carotenoids in terms of distribution in the feed, the hen’s livers and the eggs. We found that after a period of depletion, pro-vitamin A (PVA) carotenoids were preferentially diverted to the liver and relatively depleted in the eggs, whereas other carotenoids were transported to the eggs even when the liver remained depleted. When retinol was included in the diet, it accumulated more in the eggs than the livers, whereas PVA carotenoids showed the opposite profile. Our data suggest that a transport nexus from the intestinal lumen to the eggs introduces bottlenecks that cause chemically-distinct classes of carotenoids to be partitioned in different ways. This nexus model will allow us to optimize animal feed and human diets to ensure that the health benefits of carotenoids are delivered in the most effective manner.

The biofortification of staple crops with organic nutrients can help to improve nutritional health without relying on artificial fortification or supplements^1^,^2^. Examples include the biofortification of cereals such as Golden Rice producing β-carotene^3^ and Multivitamin Corn producing β-carotene, zeaxanthin, lutein, lycopene, ascorbic acid and folate^4^. Such crops not only have the potential to improve human health directly in areas predominantly reliant on cereals for nutrition^5^,^6^ but they can also improve animal health when used as feed^7^ and could pass those benefits on to humans who consume meat and other products from these animals. In the case of poultry, carotenoids in the feed are deposited in peripheral tissues to confer orange/yellow pigmentation and also into the egg yolk, which is an important source of carotenoids, particularly lutein, in the human diet^8^.

Carotenoids act as antioxidants but some are also vitamins with essential biological functions^9^,^10^,^11^. Pro-vitamin A (PVA) carotenoids such as β-carotene are converted into retinol, an essential component of the visual pigment rhodopsin that also helps to maintain epithelial and immune cells^12^. PVA carotenoids are also converted into retinoic acid, a co-regulator of developmental gene expression^13^. Vitamin A deficiency is prevalent in developing countries and is also an important health issue in developed countries, so any strategy to increase the PVA carotenoid content of food will be beneficial to society^14^. Lutein and zeaxanthin accumulate to high levels in the retinal macula and are thought to protect the macula and outer segments of the retina from oxidative stress, thus inhibiting age-related macular degeneration^15^,^16^. Vitamin A and carotenoid metabolism in chickens is closely related to the equivalent process in humans, so chickens are also susceptible to vitamin A deficiency with similar symptoms^17^,^18^. This suggests that chickens fed on carotenoid-enhanced diets may not only provide humans with nutritious meat and eggs, but may also offer good models for the behavior of carotenoids in humans.

Carotenoids are used as poultry feed additives in order to achieve the characteristic yellow-orange color of egg yolks, and typical additives include synthetic carotenoids and carotenoid-rich extracts such as marigold and red pepper^19^. The replacement of synthetic carotenoids and extracts with biofortified cereals could significantly reduce the costs of poultry feed while improving the health of chickens and passing those nutritional benefits to humans^7^. However, biofortification research has focused mainly on the accumulation of nutrients *in planta* and not on the fate of those nutrients after consumption, even though the bioaccessibility and bioavailability of nutrients in staple crops provides a more accurate indicator of nutritional quality than the nutrient content alone^20^,^21^. Current feed supplements have a major impact on the downstream benefits of poultry products because egg yolks contain predominantly lutein and are poor sources of PVA carotenoids such as β-carotene, presumably because the latter are utilized by the hen^22^. A detailed analysis of carotenoid uptake, metabolism and distribution could therefore be used to tailor poultry diets to optimize chicken health and the nutritional properties of poultry products.

In the context of human health, both PVA and non-PVA carotenoids enhance the immune system^23^. Acute infections reduce the levels of retinol and carotenoids in the plasma, particularly in infants and the elderly, suggesting that there is a higher demand for carotenoids when the body is threatened by pathogens^24^. Accordingly, a higher dietary intake of β-carotene is associated with the lower occurrence of acute respiratory infections^25^. Furthermore zeaxanthin and other carotenoids are thought to boost the production of immunoglobulin M in Th1 cells that make contact with unprimed spleen cells^26^. A recent meta-analysis concluded that vitamin A supplements reduce all-cause mortality in HIV-positive children and help to reduce diarrhea and respiratory morbidity^27^. A higher intake of carotenoids in mice fed on high-carotenoid maize diets may also offer protection against hepatomegaly and hepatic steatosis^28^.

To investigate the bioavailability and bioaccessibility of carotenoids in poultry diets and the potential consequences of different dietary interventions in terms of carotenoid availability in poultry products, we supplemented the diets of laying hens with standard maize plus a retinol supplement or two biofortified maize varieties with different carotenoid profiles. We measured the carotenoid levels in the feed, serum and liver of the hens, and in their egg yolks. We found that the uptake, distribution, metabolism and deposition of carotenoids was dependent on a range of factors that had a different impact on each type of carotenoid, such that some carotenoids were relatively depleted in the egg compared to the liver (e.g. β-carotene) whereas others were deposited in the egg against a concentration gradient, suggesting a specific transport mechanism (e.g. zeaxanthin). Our data indicate that the fate of carotenoids in animal models can be used for the development of strategies to maximize the nutritional benefits of agricultural products such as eggs. Carotenoids provided in biofortified cereals also appear more accessible than retinol supplements in a commercial diet, which may provide a direct benefit to human consumers.

## Results

### Diets containing biofortified maize provide qualitatively and quantitatively distinct carotenoid profiles compared to a commercial diet

**Figure 1:**
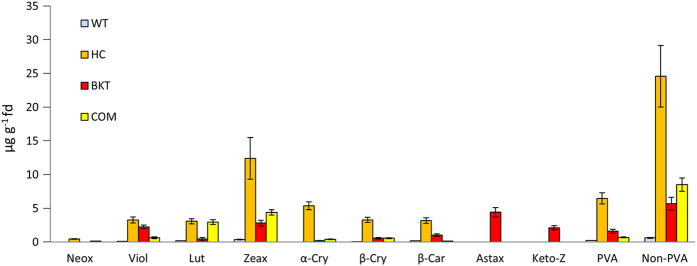
Carotenoid levels in the four diets used in the feeding trial presented as mean values in μg/g freeze dried (fd) ± SE from five samples (biological replicates). WT = wild-type M37W maize, HC = high-carotenoid maize, BKT = ketocarotenoid maize, COM = commercial yellow maize plus retinol. Individual carotenoids are shown (neox = neoxanthin, viol = violoxanthin, lut = lutein, zeax = zeaxanthin, α-cry = α-cryptoxanthin, β-cry =β-cryptoxanthin, β-car = β-carotene, astax = astaxanthin, keto-Z = other ketocarotenoids) as well as the total levels of pro-vitamin A (PVA) and non-PVA carotenoids.

In order to compare the distribution of carotenoids in hens and their eggs following the consumption of diets differing solely in terms of the carotenoid profile (carotenoid types and quantities) and the manner of presentation (supplement or biofortified maize) we prepared four different diets as shown in Table 1. All four diets contained ~62% maize. The standard commercial diet (COM) was based on a commercial yellow maize variety and was not supplemented with additional carotenoids but did contain supplemental vitamin A as is the norm in commercial poultry production. The control diet (WT) was based on the wild-type white maize variety M37W which contains only traces of carotenoids (primarily lutein and zeaxanthin) and no additional supplements were provided. The other diets were based on the biofortified maize varieties HC and BKT. The HC variety is enriched for five key carotenoids (β-carotene, lutein, zeaxanthin, α-cryptoxanthin and β-cryptoxanthin) and also contains higher levels of violaxanthin and neoxanthin than the other three maize varieties. The BKT variety is the only one to include ketocarotenoids, and is particularly enriched in astaxanthin, as well as containing relatively high levels of violaxanthin and β-carotene. The carotenoid profiles of the four diets were confirmed by HPLC analysis and are shown in Fig. 1 and Supplementary Table 1.

### Diets containing biofortified maize improve the feed conversion ratio

We assigned 32 ISA Bovans Brown Leghorn hens (33 weeks old) into four groups of eight birds which were housed in separate pens, and initially fed all four groups on the WT diet for 12 days to ensure that they all began the feeding trial in a carotenoid depleted state. After 12 days on the WT diet, three of the groups were switched to carotenoid-enriched diets (COM, HC and BKT) whereas the control group was maintained on the depleted diet. The feeding study continued for another 20 days. Eggs were collected throughout the testing period and carotenoid levels were measured at regular intervals.

We calculated the feed conversion ratio for each diet to determine whether there were any general effects that influenced the uptake and metabolism of carotenoids. The average amount of feed consumed per group of eight hens per day during the feeding trial was 925 g (WT), 744 g (HC), 870 g (BKT) and 916 g (COM). The feed conversion ratio is defined as the mass of feed consumed divided by the mass of the eggs produced and lower ratios represent greater feed conversion efficiency. We observed no differences among the treatments in terms of hen weight during the trial, although all animals lost weight during the experiment probably due to acclimatization and changes in the diet (WT = 7.0%, HC = 11.4%, BKT = 8.7%, COM = 9.3%). The feed conversion ratios for the four diets were 2.22 (WT), 1.79 (HC), 1.94 (BKT) and 2.06 (COM). Interestingly, hens fed on the HC diet produced the lowest egg mass (63 g) but also consumed the least amount of feed, resulting in the greatest feed conversion efficiency among the four diets. The BKT diet also resulted in a lower feed conversion ratio than the COM diet, whereas the WT diet was the least efficient, requiring more feed than the other diets to produce the same overall egg mass.

### The distribution of carotenoids in hens is only partially dependent on the feed profiles

We compared the carotenoid levels in eggs laid by hens after 12 days (i.e. at the end of the universal depletion period, just before the feeding trial) and after 31 days (the end of the feeding trial). We found that most carotenoids were depleted to their minimum levels after 12 days and did not decline any further when the WT diet was maintained, thus confirming that a baseline had been reached for comparison with the other three diets. However, the levels of α-cryptoxanthin declined from 0.07 μg/g (day 12) to 0.03 μg/g (day 31), lutein levels fell from 1.29 to 1.0 μg/g over the same period, and zeaxanthin likewise declined from 1.18 to 0.6 μg/g. Although these three carotenoids were not fully depleted even after 12 days on a low-carotenoid diet, the levels on day 12 were sufficient to use as a baseline for comparative analysis and is longer than the depletion period applied in a similar feeding trial^29^.

**Figure 2:**
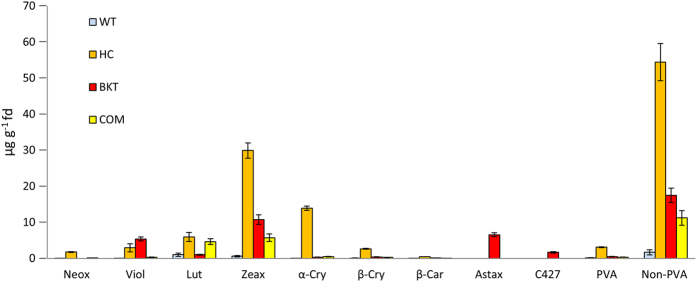
Carotenoid levels in the yolk of eggs laid by hens after 31 days on four different diets (WT = wild-type M37W maize, HC = high-carotenoid maize, BKT = ketocarotenoid maize, COM = commercial yellow maize plus retinol). Values are means in μg/g freeze dried (fd) ± SE from 3–5 independent samples (biological replicates). Individual carotenoids are shown (neox = neoxanthin, viol = violoxanthin, lut = lutein, zeax = zeaxanthin, α-cry = α-cryptoxanthin, β-cry = β-cryptoxanthin, β-car = β-carotene, C427 = most likely β-cryptoxanthin-5,8-epoxide, astax = astaxanthin) as well as the total levels of pro-vitamin A (PVA) and non-PVA carotenoids.

Because the HC and BKT diets contained two largely non-overlapping groups of carotenoids, we compared each in turn with the COM and WT diets to determine how the distribution of carotenoids in the feed (Fig. 1) compared to the distribution of carotenoids in the eggs (Fig. 2 and Supplementary Table 2). The basis for this comparison was a nexus hypothesis, in which carotenoids would be expected to accumulate in the eggs in similar proportions to their representation in the feed unless specific factors in the intercrossing transport pathways en route to the eggs resulted in either relative depletion or relative enrichment.

First, we compared the total carotenoid pool, which for the HC, COM and WT diets was 31.05, 9.22 and 0.84 μg/g, respectively, in the feed, and 57.5, 11.54 and 1.81 μg/g, respectively, in the eggs. These data suggest that carotenoids as a whole are preferentially transferred to the egg against a concentration gradient so they accumulate at higher levels in the egg than the availability in the feed would predict. However, there was a much greater proportional enrichment in the eggs when the HC diet was used instead of the COM diet. The WT diet, which is low in carotenoids, also resulted in more than double the expected concentration of carotenoids in the eggs.

To investigate the chemical and metabolic basis of these observations, we repeated the analysis separately on PVA and non-PVA carotenoids. The PVA carotenoid pool in the HC, COM and WT diets was 6.47, 0.68 and 0.23 μg/g, respectively, whereas that in the eggs was 3.09, 0.33 and 0.13 μg/g, respectively. In contrast, the non-PVA carotenoid pool in the HC, COM and WT diets was 24.58, 8.54 and 0.61 μg/g, respectively, and that in the eggs was 54.41, 11.21 and 1.68 μg/g, respectively. These data clearly show a distinction between PVA and non-PVA carotenoids, with the former showing a relative depletion of more than 50% during transfer from feed to egg and the latter showing a large enhancement, which as above was more prevalent in the HC and WT diets than the COM diet. This suggests the nexus separately channels PVA carotenoids, which are depleted on their way to the eggs, and non-PVA carotenoids, which are transported against a concentration gradient and accumulate in the eggs at up to double the concentration present in the feed, particularly in the case of the HC diet.

Similar comparative analysis of the BKT, COM and WT diets revealed that the total carotenoid pool in the BKT, COM and WT diets was 13.81, 9.22 and 0.84 μg/g, respectively, and that in the eggs was 26.18, 11.54 and 1.81 μg/g, respectively. These data again suggest that carotenoids are preferentially transferred to the egg against a concentration gradient and the enrichment is more profound when using the biofortified maize diet rather than the COM diet. Separate analysis of the PVA and non-PVA carotenoids revealed a similar profile to that described above. The PVA carotenoid pool in the BKT, COM and WT diets was 1.6, 0.68 and 0.23 μg/g, respectively, and that in the eggs was 0.46, 0.33 and 0.13 μg/g, respectively. The non-PVA carotenoid pool in the BKT, COM and WT diets was 5.7, 8.54 and 0.61 μg/g, respectively, whereas that in the eggs was 17.47, 11.21 and 1.68 μg/g, respectively. Again this suggests that non-PVA carotenoids are strongly enriched en route from the feed to the egg, particularly when supplied as a biofortified diet, whereas PVA carotenoids are depleted by up to 50% en route to the egg.

### The distribution of PVA carotenoids reflects the rate of conversion to retinol

**Figure 3:**
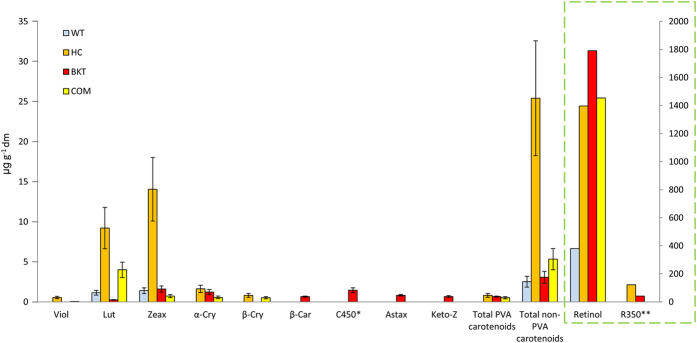
Carotenoid and retinoid levels in the livers of hens after 31 days on four different diets (WT = wild-type M37W maize, HC = high-carotenoid maize, BKT = ketocarotenoid maize, COM = commercial yellow maize plus retinol). Values are means μg/g freeze dried (fd) ± SE from five independent samples (biological replicates). Individual carotenoids are shown using the scale on the left axis (viol = violoxanthin, lut = lutein, zeax = zeaxanthin, α-cry = α-cryptoxanthin, β-cry = β-cryptoxanthin, β-car = β-carotene, C450 = most likely β-carotene-5,6-epoxide or β-carotene-5,6,5′,6′-diepoxide, astax = astaxanthin, keto-Z = other ketocarotenoids) as well as the total levels of pro-vitamin A (PVA) and non-PVA carotenoids. Retinoids are shown using the scale on the right axis and are isolated in a box for clarity. R350 is a non-polar retinoid product with an absorbance spectrum showing three distinct peaks at 328, 348, 370 nm.

One potential explanation for the relative depletion of PVA carotenoids in the eggs compared to the feed is the conversion of PVA carotenoids into retinol and its diversion to the liver. We therefore compared the levels of retinol in the livers of hens fed on the HC, BKT and WT diets to determine whether the depletion of PVA carotenoids in the egg could be explained by conversion to retinol (Fig. 3 and Supplementary Table 3). Hens fed on the COM diet were also tested but the COM diet is supplemented with 3 mg retinol per kg feed so the comparison is not informative in the context of the fate of dietary carotenoids. The mean retinol levels in the livers of hens fed on the HC, BKT, COM and WT diets were 1397, 1790, 1454 and 380 μg/g, respectively. The retinol levels in the eggs of hens fed on the HC, BKT, COM and WT diets were 18.05, 21.08, 23.69 and 15.4 μg/g, respectively. These data show that hens fed on the HC and BKT diets accumulate much more retinol in the liver than those fed on the WT diet, and given the absence of supplementary retinol in these diets the source of this additional retinol must be the PVA carotenoids in the feed, which are converted into retinol for storage in the liver and diverted away from the egg. Relatively little retinol is channeled through the nexus and into the egg and the same holds true for unconverted PVA carotenoids, which as stated above are depleted by ~50% compared to the levels present in the feed. The levels of retinol and PVA carotenoids in the serum were below the detection threshold of our HPLC procedure indicating that the liver and egg are efficient storage tissues that remove most retinol and PVA carotenoids from the circulation.

### The distribution of carotenoids in the yolk reflects the efficiency of transport to the oocyte

The accumulation of non-PVA carotenoids in the egg against a concentration gradient suggests at the least a general mechanism to favor the accumulation of this class of molecules in the yolk and potentially even the existence of a specific transport pathway for different carotenoid species. We therefore looked at the distribution of individual non-PVA carotenoids in the feed (Fig. 1), the eggs (Fig. 2) and the liver (Fig. 3) to determine whether there is any evidence for specific transport to the oocyte.

The concentration of zeaxanthin in the HC, BKT, COM and WT diets was 12.41, 2.8, 4.41 and 0.36 μg/g, respectively. The concentration of this carotenoid in the eggs was 29.89, 10.77, 5.72 and 0.6 μg/g, respectively. Finally, its concentration in the liver was 14.04, 1.6, 0.72 and 1.39 μg/g, respectively. These data confirm that zeaxanthin is transported to the egg against a concentration gradient and the gradient is steeper for the biofortified and WT diets than the COM diet.

The concentration of lutein in the HC, BKT, COM and WT diets was 3.08, 0.45, 2.97 and 0.18 μg/g, respectively. In the eggs, the lutein concentration was 5.93, 1.01, 4.64 and 1.0 μg/g, respectively. Finally in the liver, the lutein concentration was 9.2, 0.24, 4.0 and 1.13 μg/g, respectively. These data suggest that lutein is also transported against a concentration gradient but that the gradient is shallower than that for zeaxanthin. Lutein in the biofortified diets accumulates in the egg to double the concentration in the feed, whereas zeaxanthin in the biofortified diets accumulates to more like triple the concentration in the feed. The lutein and zeaxanthin in the COM diet also accumulate against a concentration gradient but the gradient is shallower than the biofortified diets suggesting that non-PVA carotenoids supplied as an integrated component of the biofortified maize are more efficiently assimilated than the same carotenoids provided as the commercial variety.

**Figure 4:**
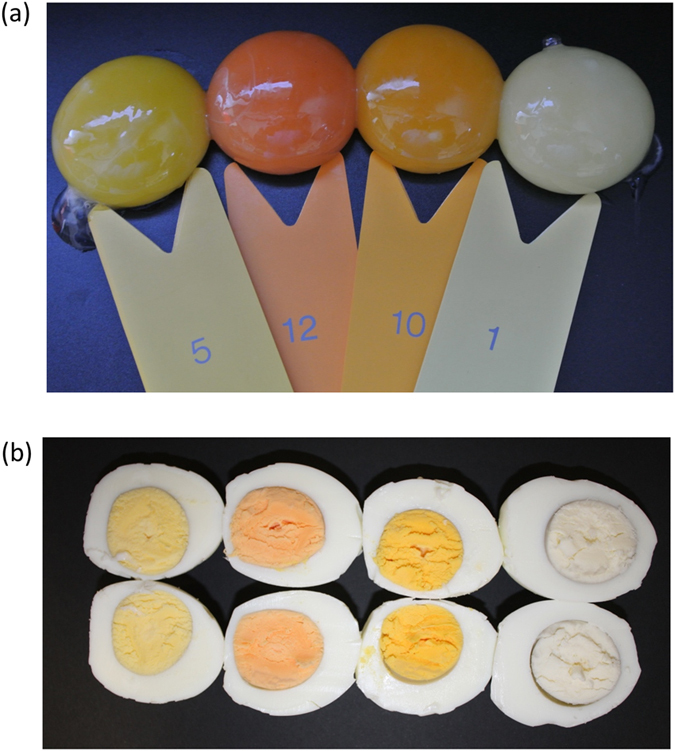
Appearance of egg yolks (**a**) raw and (**b**) cooked by boiling for 10 min produced from hens fed (left to right) on the COM, BKT, HC and WT diets. The color scale in panel (**a**) is provided by the YolkFan^TM^ (DSM N.V., Heerlen, Netherlands).

Although cross-feed comparisons were not possible with ketocarotenoids because they were only present in the BKT diet, the amount of total ketocarotenoids in the feed, eggs and liver was 6.51, 10.34 and 1.48 μg/g, respectively, whereas the corresponding values for astaxanthin, the only specific ketocarotenoid we analyzed, were 4.42, 6.56 and 0.82 μg/g, respectively. These data suggest that ketocarotenoids, and astaxanthin in particular, are also transported against a concentration gradient to accumulate in the egg. However, this is not a universal property of non-PVA carotenoids. For example, the concentration of violaxanthin in the HC, COM and WT diets was 3.25, 0.63 and 0.07 μg/g, respectively, but in the eggs the corresponding values were 2.91, 0.31 and 0.03 μg/g, respectively, indicating that violaxanthin is relatively depleted in the eggs when hens are fed on these diets. Surprisingly, the concentration of violaxanthin in the BKT feed was 2.25 μg/g but this increased to 5.38 μg/g in the eggs, indicating transport against a concentration gradient for this specific diet alone. It is therefore possible that some unique feature of this diet increases the concentration of violaxanthin in the eggs, e.g. the additional ketocarotenoids may protect violaxanthin from oxidative degradation. The ability of the eggs to accumulate specific carotenoids in the yolks was confirmed by the comparative analysis of eggs from hens fed on different diets, which showed a range of yolk colors varying from pale yellow through various hues of orange-yellow to pink-red for the yoks containing ketocarotenoids (Fig. 4).

## Discussion

Carotenoids are important organic nutrients in the human diet. The PVA carotenoids are essential precursors of vitamin A^9^,^10^,^11^ and many other carotenoids are health-promoting antioxidants, in some cases with specific roles due to their localized accumulation, such as lutein and zeaxanthin which prevent oxidative stress in the retina^15^,^16^. Although the consumption of foods rich in carotenoids (especially PVA carotenoids) is recommended to maintain nutritional health, most studies have focused on the content of food rather than the bioaccessibility and bioavailability of nutritional components. The benefits of foods containing micronutrients depend not only on the nutritional content, but also on the way it is presented (e.g. as supplements, additives or as part of the food matrix) because this affects the accessibility, solubility and potential for absorption in the gastrointestinal tract. It is difficult to design ethical tests with human subjects to study these processes in detail, so animal models are required^30^,^31^. All vertebrates absorb carotenoids using broadly similar mechanisms but there is an immense range in the ability to absorb different dietary carotenoids, and a model matching human characteristics must therefore be selected with care.

Vitamin A and carotenoid metabolism is similar in chickens and humans^17^,^18^ even though chickens convert PVA carotenoids into retinol more efficiently^32^, so chickens can be used as models for the impact of carotenoid-enhanced diets in humans. We therefore tested the impact of poultry diets based on biofortified maize enriched for different types of carotenoids on the fate and distribution of particular carotenoids in specific tissues. We fed four groups of laying hens on a carotenoid-depleted diet for 12 days and then switched three of the groups to different diets, one of which was a commercial standard diet based on yellow maize supplemented with 3 mg/kg retinol^17^ whereas the other two were based on biofortified maize varieties. We compared the proportional distribution of different carotenoids in the feed, liver and eggs.

There was a clear distinction between PVA and non-PVA carotenoids in terms of their relative distribution between the feed, liver and eggs. Compared to the profile in the feed, the PVA carotenoids (which can be converted into retinal, and then into retinol for storage) were relatively depleted in the egg and even more depleted in the liver, whereas the non-PVA carotenoids (which cannot be converted into retinol) were relatively enriched in the egg and proportionally similar in the feed and liver. This suggested the existence of some form of transport nexus from the intestinal lumen to the egg, which enhanced the accumulation of some carotenoids while diverting others. The enhancing mechanism was operative for several different chemical classes of non-PVA carotenoids, including non-PVA carotenes, ketocarotenoids and other xanthophylls, although the non-PVA carotenes and non-ketolated xanthophylls were enriched more than the ketocarotenoids when hens were fed on the BKT diet (the only diet which contained all three classes of carotenoid molecules).

An important distinction between the commercial and biofortified diets was that non-PVA carotenoids in the biofortified diets were transferred more efficiently to the egg than the same carotenoids in the commercial diet. This may reflect the fact that the commercial diet is supplemented with retinol, which may compete with the carotenoids for uptake and for other components of the shared transport nexus. The competition for carotenoid uptake begins in the stomach, where carotenoids are solubilized in lipid globules and the carotenes partition to the triacylglycerol-rich core whereas the xanthophylls stay near the monolayer surface^33^,^34^. There is a limited time for this process to occur, because surfactant bile acids in the duodenum reduce the size of the lipid droplets resulting in the formation of micelles, and although carotenoids can still move from the food matrix into the lipid phase at this point, the movement is inhibited by micelles already present in the duodenal lumen^35^. The extent to which carotenoids are taken into the micelles depends on the polarity of the carotenoid and the fatty acid composition, chain length and saturation, which would be determined by other components in the diet and by the intrinsic properties of the host organism^33^,^36^,^37^. Retinol present as a supplement might have an effect on this equilibrium, so the retinol supplement in the commercial diet could affect the uptake of carotenoids into lipid droplets.

Once carotenoids and retinol have been released from the food matrix they are taken up directly from the lumen into the enterocyte, and there may be competition among different types of molecules for receptors at this stage. Carotenoids can diffuse passively into enterocytes or can be actively absorbed by scavenger receptors such as SCARB1 and CD36^33^,^38^,^39^. Importantly, the presence of phospholipids in carotenoid micelles can inhibit their uptake^40^ which suggests that co-presented lipids in the diet, or natural differences between hosts in terms of the phospholipid content of the gut, could interfere with the absorption of carotenoids released from maize. More lipophilic carotenoids are taken up most efficiently^40^ but we observed similar enrichment in the eggs of both lipophilic and more polar carotenoids, suggesting this preferred uptake is not a rate-limiting step in the transport nexus to the egg. This effect has already been adapted to improve the efficiency of poultry feed, e.g. by supplementing the diet with lysolecithin to convert phospholipids into lysophospholipids that do not interfere with carotenoid absorption^40^,^41^. The effect of retinol supplements in the diet may also depend on the availability of cellular retinol-binding protein 2 (CRBP II) which absorbs retinol and converts it to retinyl esters for incorporation into chylomicrons (in humans) or portomicrons (in birds) for export from the gut^42^,^43^.

Within the enterocytes, carotenoids are assembled in chylomicrons/portomicrons ready for transport through the bloodstream to target organs, and carotenoids that are not assembled into such structures eventually re-enter the gut when dying enterocytes are shed into the lumen^33^. The difference between the abundance of PVA and non-PVA carotenoids we observed in the eggs probably reflects the branching of the nexus at this point, such that PVA carotenoids are diverted to the liver for storage as retinol whereas non-PVA carotenoids remain in circulating chylomicrons/portomicrons^44^. The bioconversion of PVA carotenoids into retinol requires 24 μg β-cryptoxanthin or 12 μg β-carotene for 1 μg retinol^45^.

Different types of carotenoids preferentially assemble in different lipoprotein vesicles, which are sequestered by different target organs. In the fed state, the liver will store or secrete carotenoids in very low density lipoproteins (VLDLs) and low density lipoproteins (LDLs). In the fasted state, plasma carotenes are found in LDLs, whereas the more polar carotenoids (xanthophylls) are found mainly in LDLs and high density lipoproteins (HDLs) but also to a minor extent in VLDLs. These triacylglycerol-rich lipoproteins serve as carotenoid transporters in the blood, with 55% of carotenoids in LDLs, 31% in HDLs and 14% in VLDLs. It is likely that VLDLs enriched in non-PVA carotenoids are preferentially taken up by the yolk, hence the designation VLDLy^34^. This could contribute to the observed preferential accumulation of non-PVA carotenoids and especially the preferential enrichment of the xanthophylls lutein and zeaxanthin.

Retinol is transported to the egg as a complex with retinol-binding protein (RBP) and transthyretin^46^. In the fasting state, retinol levels are usually diet-independent because retinol-RBP complexes are released from hepatic stores, whereas in the non-fasting state, the liver will store retinol provided in the diet and will convert PVA carotenoids into retinol^46^. The highest levels of retinol in the liver were observed with the biofortified diets, indicating the efficient conversion of PVA carotenoids to retinol in the liver, although high levels of retinol were also found in the livers of hens fed on the COM diet. However, the highest levels of retinol in the eggs were observed in the COM diet, suggesting that the transport of retinol to the eggs is more efficient when retinol is provided directly rather than in the form of PVA carotenoids, because the latter are preferentially converted to retinol in the liver. The differential accumulation of retinol in the eggs when provided directly as retinol or as PVA carotenoids could reflect bottlenecks at several points in the nexus, including the nature of the storage cell (hepatocyte or stellate cell), the chemical nature of the stored product (retinoic acid, trans-retinal or retinol) and the availability of carriers in relation to competition with carotenoids for particular lipoprotein complexes.

By comparing the retinol content of the liver and eggs of hens fed on the HC, BKT and WT diets, it was clear that the presence of PVA carotenoids in the biofortified maize varieties boosted the depleted retinol pool in the livers of hens fed initially on the WT diet, suggesting that PVA carotenoids are ‘siphoned off’ to rebalance the vitamin A metabolism of the hen and only a surplus is transported to the egg. In contrast, the egg accumulates non-PVA carotenoids against a concentration gradient even if the hen has been starved of all carotenoids for 12 days, which results in the liver reaching basal levels of all carotenoids except α-cryptoxanthin, lutein and zeaxanthin. In the WT group, the eggs continued to accumulate the small amounts of carotenoids provided by the diet even as the levels in the liver continued to fall, confirming that the egg is prioritized over the hen for at least these three molecules. PVA carotenoids might also be diverted to peripheral tissues, although the ISA Bovans Brown hybrid hen variety we used is known for its brown eggs yet pale skin and flesh, suggesting carotenoids do not accumulate in peripheral tissues. Most hens store carotenoids in the skin, flesh, feathers, keratinous structures (feet, beak) and crop, and those fed on a carotenoid-rich diet often present yellow or more deeply-colored appendages. However, in the ISA Bovans Brown variety used in this study, such peripheral carotenoids are likely to be degraded by β-carotene dioxygenase 2 (BCDO2) whereas varieties with yellow skin lack this enzyme^47^.

Further analysis at the level of individual carotenoids revealed that lutein and zeaxanthin both accumulate in the eggs against a concentration gradient (and concomitant with falling levels in the liver) but at different rates, with zeaxanthin accumulating to three times the concentration in the feed and lutein to only two times the feed concentration. The VLDLy structures synthesized by laying hens are smaller than those synthesized by immature hens and have the unique ability to translocate across the vitelline membrane and oocyte membrane or oolema^48^. Therefore only these smaller VLDLs pass through the basal lamina and between the cells of the granulosa layer, where they bind to the receptors on the oocyte plasma membrane. Vitellogenin and the small VLDL bind the 95 kDa receptor and undergo receptor-mediated endocytosis. The preferential transport of non-PVA carotenoids to the egg therefore appears to partly reflect their retention in the serum, and to partly reflect their ability to form VLDLy structures that are taken up by the oocyte. The structures have a hydrophobic core and a more polar monolayer, so polar carotenoids such as zeaxanthin and lutein are likely to be transported in greater abundance as smaller VLDLy structures due to the favorable surface to volume ratio, which may help to explain how zeaxanthin in particular is so highly enriched.

The dose of some carotenoids in the feed may also affect the efficiency of the nexus, potentially due to competition or cooperation for uptake and/or transport. For example, violaxanthin is relatively depleted in the egg when ingested as the HC diet but relatively enhanced when ingested as the BKT diet. The only difference between the diets is the presence of ketocarotenoids in the BKT diet and the presence of high levels of lutein, zeaxanthin and PVA carotenoids in the HC diet, suggesting that ketocarotenoids promote the transport of violaxanthin (e.g. by preventing the oxidative degradation of violaxanthin), or that there is competition between violaxanthin and one of the carotenoids in the HC diet for a shared transporter, with violaxanthin being outcompeted. Another aspect of chemical modification is the impact of stability: 4-ketocarotenoids are more stable against oxidation than 3-hydroxycarotenoids and β-carotene^49^. In particular, astaxanthin is more stable than zeaxanthin^50^. In leaf tissue with low levels of antioxidants, the photo-oxidation of violaxanthin was much faster than that of lutein^51^.

**Figure 5:**
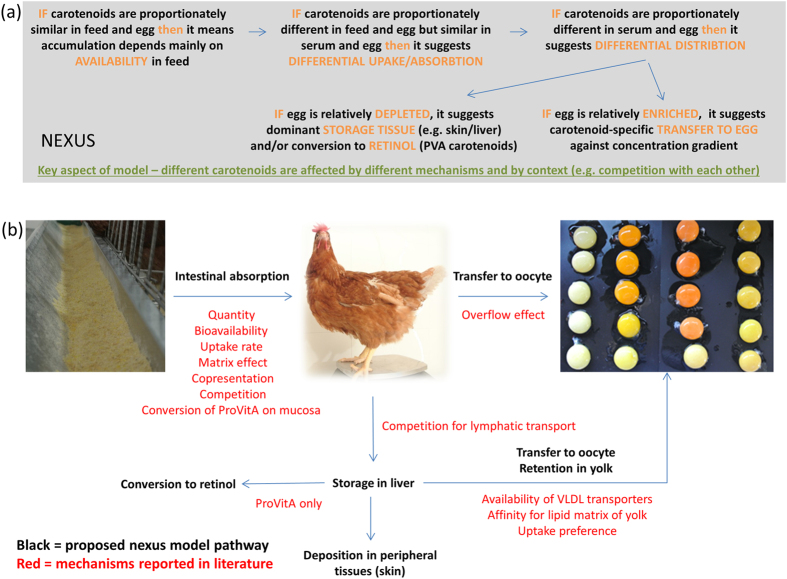
The nexus model, showing the fate of carotenoids provided to poultry as biofortified diets. (**a**) General assumptions of the model based on observations of carotenoid distribution in feed and different tissues. (**b**) The routes through the nexus (black) and mechanisms that may be involved (red) based on this study and previous reports.

These competing factors can be summed up in the overall nexus model shown in Fig. 5. The assumptions of the model are outlined in Fig. 5a, based on our observations of different carotenoid profiles in the feed and different tissues. Potential mechanisms that may generate these profiles are shown in Fig. 5b. The transport of carotenoids from the diet to the egg is influenced by multiple factors that depend on availability, accessibility, peripheral tissue demand, the chemical properties of the carotenoids, the affinity of different carotenoids for different receptors and transporters, and the ability of different lipid vesicles to cross membranes in the liver and the egg. Both the liver and the egg act as storage tissues, with the liver preferentially accumulating PVA carotenoids and the egg preferentially allocating space to non-PVA carotenoids due to a combination of utilization (the liver converts PVA carotenoids into retinol) and preferential uptake (the VLDLy vesicles preferentially cross the oolema). If these storage tissues or the transport routes leading to them become saturated, then any carotenoids not utilized directly in peripheral tissues are cleared from the body and deposited in the bird’s feces, as is also the case in humans^52^.

## Materials and Methods

### Plant material and diets

The HC and BKT transgenic lines are based on the South African elite white maize inbred M37W, which is therefore used as the wild-type control line in this study. The HC transgenic line originated from a combinatorial population of transgenic maize plants with different genotypes^53^. The HC line contains and expresses the transgenes *Zmpsy1* (*Zea mays* phytoene synthase 1) and *Pacrt*I (*Pantoea ananatis* phytoene desaturase) specifically in the endosperm. The BKT line was created by knocking down the expression of the endogenous lycopene ε-cyclase (*Zmlyce*) gene in the endosperm and expressing *Zmpsy1* (see above), the β-carotene hydroxylase (*sBrcrtZ*) transgene from the bacterium *Brevundimonas* sp. strain SD212, and a truncated β-carotene ketolase (sCrbkt) transgene from the alga *Chlamydomonas reinhardtii*^54^.

The diets were prepared at the Mas de Bover Research Centre (IRTA, Institut de Recerca i Tecnologia Agroalimentàries, Reus, Spain) and formulated according to the National Research Council^17^ updated by ISA commercial formulations (Hendrix Company, Boxmeer, Netherlands). Two diets were formulated with kernels of the biofortified maize lines HC and BKT, with enhanced levels of carotenoids and ketocarotenoids, respectively. The WT diet was formulated with kernels of the near isogenic (wild type) South African inbred white maize M37W and the COM diet with kernels of standard commercial maize supplemented with 10,000 IU vitamin A per kg (3 mg retinol/kg). The commercial maize is a mixture of different varieties grown throughout Spain.

### Feeding trial

Thirty-two laying hens (ISA Bovans Brown) were allocated in groups of eight to four diets. Hens were delivered to the Animal Research Center of the University of Lleida 33 weeks after hatching. They were weighed and placed randomly in individual cages. Prior to the commencement of the egg laying trial, all groups of hens were fed on the WT diet for the first 12 days in order to deplete or substantially reduce the amount of carotenes in the egg yolk. No retinol was added to the COM diet during the depletion phase. The diets were then changed to the experimental treatments and the subsequent trial lasted for 20 additional days. Eggs were collected daily with particular attention on days 31 and 32 to evaluate the four experimental treatments at the end of the trial. All birds were euthanized on trial day 32 for gross necropsy. Blood samples were collected for serum carotenoid analysis and pre-chilled whole livers were collected and lyophilized for carotenoid analysis. The experimental protocols were approved by the Direcció General del Medi Natural i Biodiversitat, Departament d’Agricultura, Ramaderia, Pesca, Alimentació i Medi Natural of the Generalitat de Catalunya, and were carried out in accordance with the relevant national and international guidelines and regulations.

### Carotenoid analysis

The carotenoid profiles in the different diets, egg yolks, serum and livers of hens after the trial were determined by HPLC. Total carotenoids were extracted from freeze-dried samples in 20 ml methanol containing 6% KOH at 60 °C for 15–20 min. Lipophilic compounds were partitioned into 30% ether in petroleum ether and the upper phase was collected. Total carotenoids were quantified by measuring the absorbance at 450 nm. For HPLC separation, the solvent was evaporated under a stream of nitrogen gas at 37 °C, re-dissolved in 100 μl methanol/dichloromethane (50:50), and a 20-μl aliquot was injected immediately. Compounds were separated on a 15-cm Nucleosil C18 3 μl column with an acetonitrile, methanol and 2-propanol mobile phase (85:15:5 by volume) at 20 °C. Samples were monitored with a Kontron DAD 440 photodiode array detector with online registration of the spectra. All carotenoids and ketocarotenoids were identified by co-chromatography with authentic reference compounds produced in *Escherichia coli*^55^. The standards were also used for quantification in combination with appropriate extinction coefficients^56^.

### Statistical analysis

Differences between treatments were determined by analysis of variance (ANOVA) using JMP^®^ Pro 11 (SAS, 2013). Tukey’s honest significant difference test was used for multiple means comparisons. Variables expressed in percentages were normalized using the arcsine of the square root of the probability. Data are expressed as means ± standard error and statistical significance was assumed at p < 0.05.

## Additional Information

**How to cite this article**: Moreno, J. A. *et al*. The distribution of carotenoids in hens fed on biofortified maize is influenced by feed composition, absorption, resource allocation and storage. *Sci. Rep.* **6**, 35346; doi: 10.1038/srep35346 (2016).

## Supplementary Material

Supplementary Information

## Figures and Tables

**Table 1:** Diet composition and feed analysis.

Ingredient (%)	WT^1^	HC^2^	BKT^3^	COM^4^
M37W maize	62.06	0	0	0
HC maize	0	62.06	0	0
BKT maize	0	0	62.06	0
Commercial maize	0	0	0	62.06
Soybean meal (47% protein)	25.14	25.14	25.14	25.14
Lard premium	0.54	0.54	0.54	0.54
Soybean oil	0.79	0.79	0.79	0.79
Calcium carbonate	8.64	8.64	8.64	8.64
Calcium phosphate	1.95	1.95	1.95	1.95
Sodium chloride	0.27	0.27	0.27	0.27
Sodium bicarbonate	0.20	0.20	0.20	0.20
Methionine DL	0.21	0.21	0.21	0.21
Vitamin/mineral mix^5^	0.20	0.20	0.20	0.20
Vitamin A (IU/Kg)	0	0	0	10,000

^1^WT, diet supplemented only with white maize (wild type).

^2^HC, diet supplemented with genetically engineered maize enriched in carotenoids.

^3^BKT, diet supplemented with genetically engineered maize enriched in ketocarotenoids.

^4^COM diet supplemented with standard yellow maize.

^5^Vitamin D_3_ 2000 IU/kg; vitamin E 20 IU/kg; vitamin B_1_ 2 mg/kg. vitamin B_2_ 5 mg/kg; vitamin B_6_ 2 mg/kg; vitamin B_12_ 0.02 mg/kg; vitamin K_3_ 2 mg/kg; folic acid 1 mg/kg; nicotinic acid 35 mg/kg; pantothenic acid 15 mg/kg; biotin 0.1 mg/kg; choline chloride 300 mg/kg; cooper 4 mg/kg; zinc 60 mg/kg; iron 40 mg/kg; manganese 100 mg/kg; cobalt 0.2 mg/kg; selenium 0.15 mg/kg; iodine 1 mg/kg; BHT antioxidant 4 mg/kg; anti-binder 800 mg/kg.
