# Supplementary material for: The distribution of carotenoids in hens fed on biofortified maize is influenced by feed composition, absorption, resource allocation and storage
Source: Sci Rep. 2016 Oct 14;6:35346. doi: 10.1038/srep35346 (PMC5064355; doi:10.1038/srep35346)
Supplement: Supplementary Information [file srep35346-s1.pdf]

The distribution of carotenoids in hens fed on biofortified maize is influenced by feed composition, absorption, resource allocation and storage

Jose Antonio Moreno [1,*], Joana Díaz-Gómez [1,2,*], Carmina Nogareda [1], Eduardo Angulo [1], Gerhard Sandmann [3], Manuel Portero-Otin [4], José C E Serrano[4], Richard M Twyman [5], Teresa Capell [6], Changfu Zhu [6], and Paul Christou [6,7]

Corresponding author contact details: Paul Christou, Department of Plant Production and Forestry Science, ETSEA, University of Lleida-Agrotecnio Center, Av. Alcalde Rovira Roure, 191, 25198 Lleida, Spain. Tel. +34 973 702831. E-mail christou@pvcf.udl.es

Supplementary Material

Supplementary Table 1. Feed carotenoid composition[1].

**Carotenoids**	**WT[2]**	**HC[3]**	**BKT[4]**	**COM[5]**
Neoxanthin (μg/g)	ND[6]	0.46±0.05	ND	0.12±0.01
Violaxanthin (μg/g)	0.07±0.01[a]	3.25±0.45[b]	2.25±0.27[b]	0.63±0.15[a]
Lutein (μg/g)	0.18±0.02[a]	3.08±0.38[b]	0.45±0.22[a]	2.97±0.36[b]
Zeaxanthin (μg/g)	0.36±0.03[a]	12.41±3.1[b]	2.8±0.43[a]	4.39±0.41[a]
α-Cryptoxanthin (μg/g)	ND	5.38±0.59[a]	0.2±0.03[b]	0.41±0.05[b]
β-Cryptoxanthin (μg/g)	0.03±0.01	3.29±0.39[a]	0.56±0.10[b]	0.56±0.05[b]
β-Carotene (μg/g)	0.2±0.01	3.18±0.42[a]	1.04±0.18[b]	0.12±0.02[b]
Astaxanthin (μg/g)	ND	ND	4.42±0.70	ND
Other ketocarotenoids (μg/g)	ND	ND	2.09±0.32	ND
PVA	0.23±0.01	6.47±0.81	1.60±0.28	0.68±0.07
Non-PVA	0.61±0.06	24.58±4.57	5.7±0.95	8.52±0.98
**Total**	0.84	31.05	13.81	9.2

[1] Values shown are the mean and standard error for each treatment (n = 5 biological replicates). Means within a row lacking a common superscript differ ($p < 0.05$)

[2]WT, diet supplemented only with white maize (wild type)

[3]HC, diet supplemented with genetically engineered maize enriched in carotenoids

[4]BKT, diet supplemented with genetically engineered maize enriched in ketocarotenoids

[5]COM, diet supplemented with standard commercial yellow maize

[6]ND = not detected

Supplementary Table 2. Carotenoids in egg yolks (μg/g fd) on day 32 of the trial (day 20 of experimental diet)[1].

**Carotenoid (μg/g)**	**WT[2]**	**HC[3]**	**BKT[4]**	**COM[5]**
Neoxanthin	0.02±0.01[a]	1.75±0.15[b]	ND[6]	0.09±0.02[a]
Violaxanthin	0.03±0.01[a]	2.91±1.11[b]	5.38±0.52[b]	0.31±0.07[a]
Lutein	1±0.47[a]	5.93±1.22[b]	1.01±0.11[a]	4.64±0.8[b]
Zeaxanthin	0.6±0.19[a]	29.89±2.09[b]	10.77±1.33[c]	5.72±1.03[ac]
α-Cryptoxanthin	0.03±0.01[a]	13.93±0.58[b]	0.31±0.03[a]	0.45±0.09[a]
β-Cryptoxanthin	0.08±0.02[a]	2.64±0.13[b]	0.37±0.06[a]	0.28±0.04[a]
β-Carotene	0.05±0.02[a]	0.45±0.02[b]	0.09±0.01[a]	0.05±0.01[a]
Astaxanthin	ND	ND	6.56±0.56	ND
Other ketocarotenoids	ND	ND	1.69±0.21	ND
PVA	0.13±0.04	3.09±0.15	0.46±0.07	0.33±0.05
Non-PVA	1.68±0.69	54.41±5.15	17.47.±1.99	11.21±2.01
**Total**	**1.81**	**57.5**	**26.18**	**11.54**

[1]Values shown are the mean and standard error for each treatment (n = 5 biological replicates). Means within a row lacking a common superscript differ ($p < 0.05$)

[2]WT, diet supplemented only with white maize (wild type)

[3]HC, diet supplemented with genetically engineered maize enriched in carotenoids

[4]BKT, diet supplemented with genetically engineered maize enriched in ketocarotenoids

[5]COM, diet supplemented with standard commercial yellow maize

[6] ND = not detected

Supplementary Table 3. Carotenoids in liver (µg/g fd) at day 32 (20th day of feed treatment) of the experiment.

Carotenoid	WT[2]	HC[3]	BKT[4]	COM[5]
Violaxanthin (µg/g)	ND	0.55±0.15^{a}	ND	0.05±0.01^{b}
Lutein (µg/g)	1.13±0.3^{b}	9.2±2.59^{a}	0.24±0.05^{b}	4.0±0.95^{ab}
Zeaxanthin (µg/g)	1.39±0.37^{b}	14.04±3.96^{a}	1.6±0.38^{b}	0.72±0.19^{b}
α-Cryptoxanthin (µg/g)	ND	1.62±0.45	1.22±0.32	0.56±0.16
β-Cryptoxanthin (µg/g)	ND	0.81±0.23	ND	0.51±0.14
β-Carotene (µg/g)	ND	ND	0.65±0.08	ND
C450*	ND	ND	1.45±0.31	ND
Astaxanthin (µg/g)	ND	ND	0.82±0.11	ND
Other ketocarotenoids (µg/g)	ND	ND	0.66±0.11	ND
R350**	ND	121.4±24.9^{a}	41.6±6^{b}	ND
Retinol	380±74^{b}	1397±218^{a}	1790±308^{a}	1454±236^{a}
PVA	0	0.81±0.23	0.65±0.08	0.51±0.14
Non-PVA	2.52±0.67	25.41±7.15	3.06±0.75	5.33±1.31

*C450 most likely β-carotene-5,6-epoxide or β-carotene-5,6,5',6'-diepoxide; tr trace amounts below 0.01;. *R350 is regarded as a non-polar retinoid-related product with an absorbance spectrum showing three distinct peaks at 328, 348, 370 nm

[1]Values shown are the mean and standard error for each treatment (n = 5 biological replicates). Means within a row lacking a common superscript differ ($p < 0.05$)

[2]WT, diet supplemented only with white maize (wild type)

[3]HC, diet supplemented with genetically engineered maize enriched in carotenoids

[4]BKT, diet supplemented with genetically engineered maize enriched in ketocarotenoids

[5]COM, diet supplemented with standard commercial yellow maize

[6] ND = non detected
